# Knowledge, Attitudes, and Practices Regarding Brucellosis among Parents in Aseer Region, Southwestern Saudi Arabia

**DOI:** 10.3390/healthcare9111541

**Published:** 2021-11-11

**Authors:** Youssef A. Alqahtani, Ayed A. Shati, Saleh M. Al-Qahtani, Ali A. Asseri, Ahmad A. Alhanshani, Fatimah M. Alqahtani, Afnan M. Alqarni, Mona A. Alqarni, Mohamed E. Hamid

**Affiliations:** 1 Department of Child Health, College of Medicine, King Khalid University, Abha 62529, Saudi Arabia; Yal-qahtani@kku.edu.sa (Y.A.A.); ashati@kku.edu.sa (A.A.S.); smuadi@kku.edu.sa (S.M.A.-Q.); alsoheel11@kku.edu.sa (A.A.A.); aanasir@kku.edu.sa (A.A.A.); 2 Department of Family Medicine, King Fahad Medical City, Riyadh 12231, Saudi Arabia; fatalq995@gmail.com (F.M.A.); dr.af.alqarni@gmail.com (A.M.A.); dr.monaalgarni@gmail.com (M.A.A.); 3 Department of Microbiology, College of Medicine, King Khalid University, Abha 62529, Saudi Arabia

**Keywords:** brucellosis, knowledge, altitude, practices, parents, Aseer region

## Abstract

This study aimed to investigate the knowledge, attitudes, and practices regarding brucellosis among parents in the Aseer region of southwestern Saudi Arabia in order to estimate the population sectors that are at high risk for accidental exposure to brucellosis. This was a cross-sectional survey conducted in 2018, including 311 participants representing various genders, ages, and levels of education. Bivariate analysis showed a significant association of good awareness of the disease with the male gender and higher education levels. Of the 311 participants, 90.0% had good knowledge, whereas 10.0% showed inadequate knowledge about brucellosis. Practices and attitudes regarding brucellosis were satisfactory as participants did not eat meat from their own animals (52.7%), did not practice slaughtering (71.4%), did not participate in the birth of animals (91.3%), were not exposed to abortion in animals (93.2%), and practiced burial of aborted animal fetuses (59.2%). The practice regarding brucellosis was unsatisfactory as 66.6% never wore gloves when dealing with animals. The study concluded that the majority of parents showed fair and positive knowledge, attitudes, and practices regarding brucellosis and found that gender and education were determinants of satisfactory awareness.

## 1. Introduction

Brucellosis (also called Malta fever or Mediterranean fever) is a zoonotic infection caused by bacteria in the genus *Brucella*, which cause significant health and economic loss [1]. Brucellosis is an important zoonosis and is neglected in some parts of the world [1,2,3,4]. New epidemiological aspects of brucellosis, including its emergence in new regions as well as its growing transmission from animals to humans, are of great significance [5,6].

Brucellae are Gram-negative, non-spore-forming, non-motile coccobacilli and facultative intracellular parasites that cause a chronic disease that usually persists for life [3,7]. Four common species can infect humans, including *B. abortus*, *B. melitensis*, *B. canis* and *B. suis*, and less commonly is *B. inopinata*. The symptoms of brucellosis include joint and muscle pain and sweating. The disease is transmitted from animals to humans through direct contact with infected animals, ingestion of infected food products, or inhalation of aerosols [8].

Brucellosis is endemic in most developing countries and manifests as a febrile illness that is sometimes indistinguishable from malaria or typhoid fever; hence, it may not be recognized in clinical and laboratory settings [9,10,11]. It is quite prevalent in Middle Eastern [12] and east African countries [13]. The prevalence of brucellosis in humans was found to be between 0.0% and 35.8% in East African communities [14]. The prevalence in livestock was 0.2% to 43.8%, 0.0% to 20.0%, and 0.0% to 13.8% in cattle, goats, and sheep, respectively [14].

Brucellosis is endemic in Saudi Arabia and is regarded as a major public zoonotic infection. It has been reported to occur in most regions in the Kingdom [15,16,17,18,19], including the southern region [20]. In a randomized, multistage sampling of 1200 abattoir workers throughout Saudi Arabia, brucellosis was found more commonly among butchers (8.9%), veterinarians and veterinary assistants (5.4%), and administrative personnel (1.1%) [21]. A high prevalence (12.8%) of human brucellosis was recorded in the Aseer region [22]. Human brucellosis among the selected exposed agro-pastoral communities in southern Saudi Arabia was found to have a prevalence of 53.3% in northwestern Asia compared to 25.9% in southeastern Aseer and 20.6% in the Jazan region (20.6%). The import of a large number of sheep to meet the requirements of the Hajj season is one of the important reasons for the spread of the disease in the Kingdom. Thus, studies concerned with the transmission and spread of the disease are of great importance [15].

Lack of awareness of the disease among medical practitioners, apart from that in the parents, adds to its general neglect [23]. Gender and education level were the only determinants of good awareness and safe behavior among individuals who depend on livestock as their source of income or food. Additionally, there is a high risk of zoonotic exposure due to their close contact with livestock, causing a subclinical infection that may not be diagnosed and appropriately treated [13,24]. Parents’ awareness regarding the impact of brucellosis is lacking [25,26]. Improving the awareness of parents through health education programs covering the gaps in knowledge is vital to controlling this chronic health problem. 

The current study aimed to assess the knowledge, attitudes, and practices of parents regarding brucellosis in the Aseer region, a southwestern region of Saudi Arabia, to identify the population sectors at high risk of accidental exposure to brucellosis.

## 2. Materials and Methods

### 2.1. Study Design

A descriptive, cross-sectional survey was conducted from August 2018 to December 2018 among parents in the Aseer region in southwestern Saudi Arabia. Participation was voluntary, and informed consent was obtained from all participants. An electronic questionnaire developed by the researchers after an intensive literature review and expert consultation for validity and applicability was used for data collection. Modifications were considered until the final format was determined, which was posted online through social media.

### 2.2. Questionnaire

The questionnaire covered parents’ sociodemographic data and awareness regarding brucellosis causes, mode of transmission, and clinical presentation. Furthermore, parents were asked about their behavior towards animals in terms of abortion, sharing in animal birth, and exposure to excreta. Finally, their attitudes towards consuming homemade milk or cheese and their own animals’ meat were queried.

### 2.3. Data Analysis

After the data were extracted, they were revised, coded, and fed into the statistical software IBM SPSS version 22 (SPSS, Inc., Chicago, IL, USA). Two-tailed tests were used for all statistical analyses. A *p*-value less than 0.05 was considered statistically significant. Each correct answer among the awareness items was awarded one point. The discrete scores of the awareness items were summed, and the overall score was categorized as poor awareness levels for those who had a score less than 50% of the maximum score (11 points) and good awareness levels if the score exceeded 50% of the maximum score. Descriptive analysis based on frequency and percent distribution was carried out for all variables, including demographic data, clinical data, and awareness and attitude variables. The univariant relationship between participants’ data and their awareness level was tested using the Pearson chi-square test.

## 3. Results

### 3.1. Distribution of Awareness by Parents’ Personal Data

The study included 311 parents representing different genders, ages, levels of education, occupations, and family sizes. The participants’ ages ranged from 20 to 65 years, with a mean age of 29.2 ± 9.7 years. Of the participants, 29.6% were male parents and 37.9% had families of 1–5 persons. University-level education was recorded among 79.7% of the parents (Table 1).

Overall, most parents (73.6%) had good awareness of brucellosis. Awareness varied by age group (*p* = 0.101), male parents had better awareness than female parents (*p* = 0.101), and awareness was affected by the level of education (*p* = 0.001) but not by family size (Table 1).

### 3.2. Knowledge about Brucellosis

**Figure 1:**
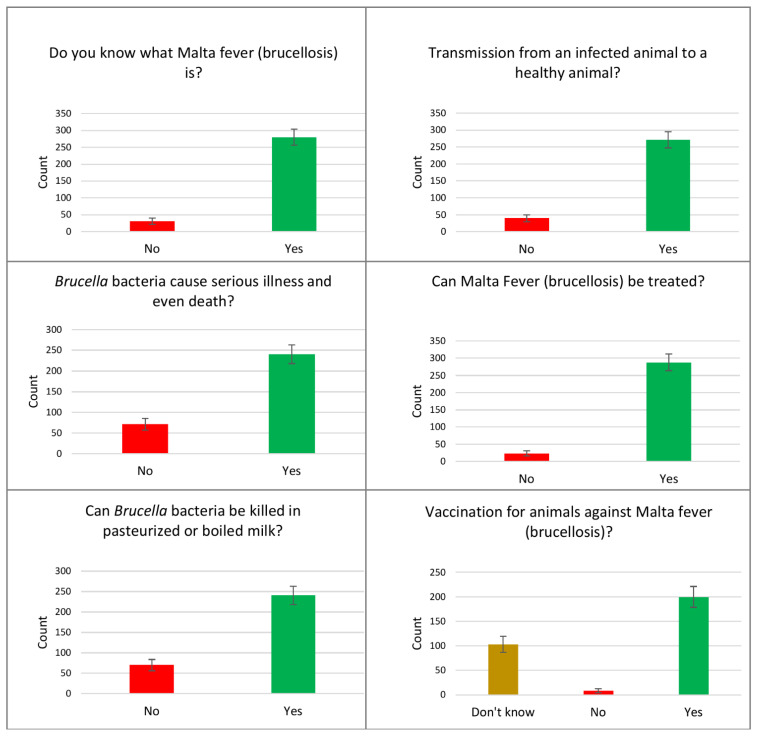
Items of awareness regarding brucellosis among parents in the Aseer region, southwestern Saudi Arabia.

Data on knowledge about brucellosis, including the disease, its source, symptoms, transmission, and management among the parents in the Aseer region of Saudi Arabia, are shown in Table 2, and some items are illustrated in Figure 1. Ninety percent (90.0%) of the study participants knew what Malta fever (brucellosis) is, and 37.9% reported that their information was derived from relatives and friends, followed by books (16.1%), multiple sources (15.8%), or TV (15.1%).

Regarding the disease, 83.6% knew which animals can be infected, 85.2% recognized humans as a susceptible species as well as the disease manifestations, 80.7% recognized the major symptoms of brucellosis, and 64.3% thought that there are vaccinations for animals with Malta fever.

A good proportion of parents gave a positive response regarding knowledge of transmissibility between animals to humans, animal to animal and human to human. When parents were asked whether brucellosis causes serious illness and even death, 77.2% said yes but the same proportion reported that the disease can be treated in humans using medicines, and that the bacteria could be killed when milk is pasteurized or boiled to a temperature of at least 63 °C (Table 2).

### 3.3. Practices and Attitudes towards Brucellosis

Data on practices (behaviors) and attitudes towards brucellosis among the parents in the Aseer region, including meat consumption, slaughtering practices, contact with animals or their fetuses, and wearing gloves are shown in Table 3.

The majority of the participants (71.4%) never practiced the slaughter of animals (52.7%) and neither participated in the birth of animals (91.3%) nor were exposed to abortion in animals (93.2%). A total of 66.6% used no gloves when dealing with animals.

Parents’ preferences were varied; homemade cheese was preferred to stumped cheese in the market by 56.6%, whereas only 15.1% saw domestic milk as better than market-traded milk (Table 3).

The practices and attitudes regarding brucellosis among parents in the Aseer region, Saudi Arabia, are shown in Table 3. The different sources of knowledge of the disease among the studied population are illustrated. Friends and relatives were the most recorded source (48.6%), followed by participants’ studies (33.4%), mass media (25.7%), medical staff (5.8%), and the internet (4.2%) (Table 2).

## 4. Discussion

The present survey-based study investigated the level of knowledge, awareness, and attitudes towards brucellosis and its health risks among parents in the Aseer region, Saudi Arabia. Awareness of any disease should translate to good overall knowledge, increasing public health education and knowledge about diseases in general [27,28], and brucellosis in particular [2,29,30,31]. Our study showed that the parents in the Aseer region were familiar with brucellosis, towards which they had average practices and a good attitude. Awareness of brucellosis among parents was good (73.6%), with male parents being more aware than female parents. This is a comparatively good level. For example, a meta-analysis was carried out to obtain pooled brucellosis awareness levels and knowledge levels of respondents regarding the zoonotic nature of brucellosis and indicated that the total pooled awareness level of brucellosis was 55.5% [2]. 

The current study assessed parents’ awareness of brucellosis in the Aseer region, Saudi Arabia. In addition, it aimed to map their attitudes and practices regarding brucellosis. It was found that three out of four participants were aware of brucellosis. The highest awareness level was recorded for methods of transmission between animals and from animals to humans, but awareness was lower regarding transmission between humans. Moreover, good awareness was recorded for brucellosis clinical manifestations and availability of treatment methods. The most important (significant) predictor for parents’ awareness level was gender. This is explained by the fact that male parents in Saudi communities carry out work, including livestock breeding, so they are more likely to have contact with animals or friends who may have some awareness. Besides this, highly educated parents were more aware, and most such parents reported studies as the second common source of information after friends. Regarding the source of knowledge, it was clear that medical staff had almost neglected their role in improving individuals’ awareness, indicating a gap in their role, especially among infectious diseases specialists and veterinarians. Brucellosis needs to be included in public awareness programs and public health education, especially in rural areas providing more knowledge and awareness to parents and their children [32]. 

Regarding practices and attitudes, the survey revealed that less than half of the parents preferred homemade food, especially meat and cheese, perhaps to avoid unsafe sources. Moreover, the percentage of parents who engaged in risky behaviors such as participating in animal birth or abortion, and exposure to excreta, were lower than expected. This can be explained by the fact that most participants had high education levels and employment capacity and did not have animals (high social level). Additionally, those who had contact with animals reported safe behavior for suspected cases in animals, including burial of the aborted embryo and separation of the suspected animals. Brucellosis is a known zoonotic infection worldwide, and awareness and knowledge of brucellosis among occupational workers are considered important aspects of brucellosis control in both humans and animals [2,33]. The disease is still an uncontrolled, and a considerable public health problem in numerous developing countries [34]. The disease may be ignored or misdiagnosed or pass undiagnosed because of challenges to diagnosis and the lack of diagnostic laboratory experience in some peripheral areas [35]. Regionally, five countries in Asia recorded the highest incidence of human brucellosis. Syria has the highest annual incidence of brucellosis worldwide, reaching 1603 cases per million per year, followed by Turkey with 15,000 cases in 2004. However, in Kuwait, the incidence is at 500 cases per million [36]. In Saudi Arabia, a national survey stated that the prevalence rate of human brucellosis reached 40 cases per 100,000. However, the incidence rate was reduced to 16.89/100,000 in 2006 [37].

Enhancing the awareness of brucellosis and brucellosis-related consequences is an important issue for its effective control [38]. Health education regarding the disease’s nature, and high-risk groups, is essential in gaining support for limiting its disease. Therefore, assessing the awareness level among parents and their children is a starting point for the development and implementation of fruitful health education programs and brucellosis control programs fitting local communities’ needs and perceptions [1,13,39].

One limitation of this study is that the sample size (female/male) did not match, since it included 219 females compared to 92 males. This could mainly be attributed to fact that females were more concerned about the study and completed the questionnaire more conscientiously, compared to males who have tendencies to overlook and ignore such thing.

## 5. Conclusions

In conclusion, the parents in the Aseer region of southwestern Saudi Arabia showed high awareness, average practices, and a good attitude regarding brucellosis and related issues. Male parents and education level were determinants of adequate awareness and safe behavior. Health education programs targeting animal breeders and others having direct contact with animals should be an area of attention for health care planners. Such programs can be disseminated through mass media announcement posters and medical staff in different health care facilities, especially primary health care centers.

The study has some limitations. Despite the efforts put into this survey, the method of data collection, which was based on an online questionnaire, is considered a limitation as it targets community members who are educated and have internet access through smartphones and are mainly residents in urban regions. This may cause bias as the target groups who deal more with animals in rural regions may not be accessible by this method. However, this explains the high awareness level recorded and can be used as an indicator for the magnitude of the problem. Another inadequacy of this survey is the fact that the sample size (n = 311) did not consider the total population in the region which is estimated as 2.212 million.

## Figures and Tables

**Table 1:** Awareness level distributed by parents’ data in Aseer region, southwestern Saudi Arabia.

Personal Data	Awareness Level				*p*-Value *
	Poor		Good		
	Number	Percent	Number	Percent	
Age in years					0.101
<25	38	28.1	97	71.9	
25–34	18	18.4	80	81.6	
35–44	19	36.5	33	63.5	
>45	7	26.9	19	73.1	
Gender					0.002
Male parents	13	14.1	79	85.9	
Female parents	69	31.5	150	68.5	
Family size					0.945
1–5	32	27.1	86	72.9	
6–10	42	26.3	118	73.8	
>10	8	24.2	25	75.8	
Education					0.001
Secondary school and below	41	78.8	11	21.2	
Diploma	2	33.3	4	66.7	
Bachelor	36	14.5	212	85.5	
Master	3	60.0	2	40.0	

P: Pearson X^2^ test; * *p* < 0.05 is significant.

**Table 2:** Items of awareness regarding brucellosis among parents in the Aseer region, southwestern Saudi Arabia.

Awareness Item (Criteria)	Response	Number	Percent	*p*-Value	95% Confidence Interval	
					Lower	Upper
Do you know what Malta fever (brucellosis) is?	No	31	10.0	0.000	1.87	1.93
	Yes	280	90.0			
Source of your information?	Books	50	16.1	0.000	5.85	6.44
	Books or TV	6	1.9			
	I do not know	4	1.3			
	In college	1	0.3			
	Internet	7	2.3			
	Multiple sources	49	15.8			
	Relatives and friends	118	37.9			
	School or university	17	5.5			
	TV	47	15.1			
	Veterinary field	12	3.9			
Which animals can be infected?	Do not know	51	16.4	0.000	3.62	3.84
	Domestic animals	190	61.1			
	Domestic plus pigs and camels	3	1.0			
	All mammals	67	21.5			
Can a human become infected?	No	46	14.8	0.000	3.68	3.82
	Yes	265	85.2			
What are the symptoms (manifestations) in humans?	Do not know	30	9.5	0.000	5.60	5.86
	Arthritis	114	36.8			
	Fever	112	36.2			
	Malaise	39	12.6			
	Skin manifestations	16	5.0			
Transmission from an infected animal to a healthy animal?	No	40	12.9	0.000	2.83	2.91
	Yes	271	87.1			
Transmission from an infected animal to a healthy human?	No	31	10.0	0.000	2.87	2.93
	Yes	280	90.0			
Transmission from an infected human to a healthy human?	No	136	43.7	0.000	2.51	2.62
	Yes	175	56.3			
*Brucella* bacteria cause serious illness and even death?	No	71	22.8	0.000	2.72	2.82
	Yes	240	77.2			
Can Malta Fever (brucellosis) be treated?	No	23	7.4	0.000	2.90	2.96
	Yes	288	92.6			
Can *Brucella* bacteria be killed in pasteurized or boiled milk?	No	70	22.5	0.000	2.73	2.82
	Yes	241	77.5			
Vaccination for animals against Malta fever (brucellosis)?	Do not know	103	33.1	0.000	2.21	2.42
	Yes	200	64.3			
	No	8	2.6			

**Table 3:** Practices and attitudes regarding brucellosis among parents in the Aseer region, southwestern Saudi Arabia.

Practice and Attitude Item (Criteria)	Response	Number	Percent	*p*-Value	95% Confidence Interval	
					Lower	Upper
Eating meat from my animals?	Never	164	52.7	0.000	3.14	3.28
	Sometimes	106	34.1			
	Usually	41	13.2			
Slaughter of my own animals?	Never	222	71.4	0.000	3.12	3.23
	Sometimes	72	23.2			
	Always	17	5.5			
Participate in the birth of animals?	Never	284	91.3	0.000	3.00	3.07
	Sometimes	19	6.1			
	Always	8	2.6			
Use gloves when dealing with animals?	Never	207	66.6	0.000	2.87	3.00
	Sometimes	42	13.5			
	Always	62	19.9			
Exposed to abortion in animals?	Never	290	93.2	0.000	2.99	3.05
	Sometimes	13	4.2			
	Always	8	2.6			
What do you do with the aborted animal fetus?	Do not know	27	8.7	0.000	5.40	6.19
	Burial	184	59.2			
	Give to dogs	46	14.8			
	Throw it on the banks of the canals	22	7.1			
	Burning	29	9.3			
	Throw it in the junk	3	1.0			
What do you do with an animal after a miscarriage?	Do not know	32	10.3	0.000	8.48	9.22
	Keep it	66	21.2			
	Separate it from others	167	53.7			
	Selling	18	5.8			
	Slaughter	28	9.0			
Homemade cheese better than market cheese?	Never	176	56.6	0.000	3.07	3.21
	Sometimes	89	28.6			
	Always	46	14.8			
Domestic milk is the best?	Never	166	53.4	0.000	2.09	2.23
	Sometimes	98	31.5			
	Always	47	15.1			

## Data Availability

The datasets produced or analyzed during the current study are available from the corresponding author on request.
